# Comparative Effects of Myo-Inositol, Metformin, Berberine, and Combination Therapy on Metabolic, Anthropometric and Hormonal Parameters in Women with Polycystic Ovary Syndrome: A 6-Month Observational Cohort Study

**DOI:** 10.3390/jcm15207765

**Published:** 2026-10-09

**Authors:** Vesselina Yanachkova, Angel Yordanov, Antonia Yaneva, Hristina Lebanova, Svetoslav Stoev

**Affiliations:** 1 Research Institute, Medical University-Pleven, 5800 Pleven, Bulgaria; v_ess@abv.bg; 2 Department of Endocrinology, Specialized Hospital for Active Treatment of Obstetrics and Gynecology “Dr. Shterev”, 1330 Sofia, Bulgaria; 3 Department of Gynecologic Oncology, Medical University-Pleven, 5800 Pleven, Bulgaria; 4 Department of Medical Informatics, Biostatistics and E-learning, Faculty of Public Health, Medical University-Plovdiv, 4002 Plovdiv, Bulgaria; 5 Department of Pharmaceutical Sciences and Social Pharmacy, Faculty of Pharmacy, Medical University-Pleven, 5800 Pleven, Bulgaria

**Keywords:** polycystic ovary syndrome, PCOS, insulin resistance, metformin, myo-inositol, berberine, metabolic outcomes, hyperandrogenism

## Abstract

**Background:** Polycystic ovary syndrome (PCOS) is the most prevalent endocrine condition among women of reproductive age. It is marked by metabolic and reproductive irregularities and is often linked to insulin resistance, dyslipidemia, and obesity. Diverse methods of treatment are employed to enhance metabolic and hormonal parameters, including inositol-based supplements, metformin, and novel compounds such as berberine. **Objective:** To describe changes observed over six months in anthropometric, metabolic, lipid, and hormonal metrics among women with PCOS undergoing myo-inositol, metformin, berberine, or a combination of metformin and berberine in standard clinical settings, and to investigate if disparities among treatment groups remained after controlling for initial characteristics. **Materials and Methods:** This retrospective observational cohort study included 148 women with PCOS diagnosed according to the Rotterdam criteria who completed six months of treatment with myo-inositol (*n* = 38), metformin (*n* = 52), berberine (*n* = 28), or combined metformin–berberine therapy (*n* = 30). Anthropometric, metabolic, lipid, and androgen parameters were assessed at baseline and after six months. Within-group changes were evaluated with correction for multiple testing using the Holm method. Given the non-random treatment allocation and baseline differences among groups, supplementary baseline-adjusted analyses were performed for the continuous outcomes. **Results:** Treatment groups differed significantly in several baseline metabolic variables including body mass index (BMI), fasting plasma glucose (FPG), fasting insulin and homeostatic model assessment of insulin resistance (HOMA-IR). After Holm correction for multiple testing, the metformin group showed significant reductions in BMI, FPG, markers of insulin resistance, lipid parameters, and androgen-related hormonal parameters, together with an increase in high-density lipoprotein cholesterol (HDL-C). In the myo-inositol group, significant reductions were observed in fasting insulin, HOMA-IR, total cholesterol, low-density lipoprotein cholesterol (LDL-C), triglycerides, androstenedione, and dehydroepiandrosterone sulfate (DHEAS). In the berberine group, significant reductions were observed in BMI, fasting insulin, HOMA-IR, total cholesterol, triglycerides, and androstenedione. In the combined metformin–berberine group, significant reductions were observed in BMI, fasting insulin, HOMA-IR, total cholesterol, LDL-C, triglycerides, testosterone, androstenedione, DHEAS, and 17-hydroxyprogesterone, together with an increase in HDL-C. In the baseline-adjusted analyses, no significant overall treatment-group effects remained for the principal metabolic outcomes after correction for multiple testing, whereas significant overall treatment-group effects were observed for DHEAS and 17-hydroxyprogesterone. **Conclusions:** In this real-world observational cohort, six months of treatment with metformin, myo-inositol, berberine, or combined metformin–berberine therapy was associated with changes in several metabolic and hormonal parameters. However, substantial baseline differences among the clinically selected treatment groups and the absence of significant baseline-adjusted between-group effects for the principal metabolic outcomes preclude conclusions regarding comparative treatment efficacy. The observed between-group differences in selected hormonal outcomes warrant further investigation in prospective comparative studies.

## 1. Introduction

Polycystic ovary syndrome (PCOS) is the most common endocrine disorder affecting women of reproductive age, with a prevalence ranging from 6% to 20%, depending on the diagnostic criteria applied. The diagnosis of PCOS is most commonly established according to the Rotterdam criteria, endorsed by the European Society of Human Reproduction and Embryology (ESHRE) and the American Society for Reproductive Medicine (ASRM). These criteria require the presence of at least two of the following three features: oligo- or anovulation, clinical and/or biochemical hyperandrogenism, and polycystic ovarian morphology on ultrasound, after exclusion of other etiologies [1,2,3,4].

PCOS is a heterogeneous disorder with recognized phenotypic variation in its reproductive, androgenic, and metabolic manifestations [5,6,7,8,9]. Although this phenotypic heterogeneity provides relevant clinical context, individual PCOS phenotypes were not evaluated in the present study and were not used for treatment allocation or analysis of treatment response. In recent years, the clarification of the role of metabolic disorders in the pathogenesis of the disease has also called into question the traditional name “polycystic ovary syndrome” [10]. The term polycystic ovary syndrome (PCOS) has traditionally been used in international evidence-based guidelines, including the 2023 International Evidence-based Guideline for the Assessment and Management of PCOS [4]. More recently, following a multistep global consensus process, “Polyendocrine Metabolic Ovarian Syndrome” (PMOS) was adopted as the new name for the condition, reflecting its broader endocrine, metabolic, and ovarian features [11]. Given that the participants in the present study were diagnosed and managed within the established PCOS diagnostic framework, the term PCOS is retained throughout this manuscript for consistency with the diagnostic criteria applied to the study population.

Insulin resistance and the subsequent compensatory hyperinsulinemia are one of the main pathophysiological mechanisms in the development of polycystic ovary syndrome. Hyperinsulinemia stimulates ovarian androgen production, suppresses the synthesis of sex hormone-binding globulin (SHBG) in the liver, and thus contributes to the development and maintenance of hyperandrogenism, one of the characteristics of the syndrome. In addition, disorders in insulin signaling have a significant impact on glucose metabolism, adipose tissue, and energy balance, which determines the increased risk of metabolic complications in these patients [9,10,11,12]. In this regard, metabolic risk assessment should be an integral part of the diagnostic process in all patients with PCOS regardless of their phenotype.

The clinical significance of these mechanisms is determined by the numerous short-term and long-term consequences of the disease. In addition to reproductive disorders, the syndrome is associated with an increased risk of type 2 diabetes mellitus, metabolic syndrome, cardiovascular diseases, obstructive sleep apnea, and endometrial carcinoma, as well as various psychoemotional disorders, including depression and anxiety [4,13]. Due to the central role of insulin resistance in the pathogenesis of the disease, therapeutic strategies aimed at improving insulin sensitivity occupy a key place in the modern treatment of the syndrome. In addition to the widely used metformin, substances such as myo-inositol, which is involved in insulin signaling, and berberine, whose metabolic effects include improving glucose homeostasis, reducing insulin resistance, and favorably affecting the lipid profile, are of increasing interest [14].

For decades, metformin has remained the most widely used insulin-sensitizing drug in patients with PCOS. As a member of the biguanide group, it reduces hepatic glucose production by partially inhibiting mitochondrial respiratory complex I and improves peripheral glucose uptake. As a result, metformin reduces hyperinsulinemia, improves insulin sensitivity, and indirectly affects hyperandrogenism. Clinical studies have shown that metformin treatment can improve menstrual cycle, ovulatory function, and some metabolic parameters, especially in patients with proven insulin resistance [15].

Despite its proven efficacy, metformin use is associated with several limitations. The most common adverse drug reactions include gastrointestinal complaints such as nausea, abdominal discomfort, diarrhea, and flatulence, which can lead to reduced adherence to therapy. These limitations stimulate the search for alternative or complementary therapeutic agents with a better tolerability profile.

In recent years, myo-inositol has attracted considerable interest. It is a natural cyclic polyol that is involved in intracellular insulin signaling processes, acting as a second messenger in a number of hormonal pathways. Data show that its administration can improve insulin sensitivity, restore ovulatory function, and also have a beneficial effect on the metabolic profile of patients with PCOS. Myo-inositol is characterized by very good tolerability and a low risk of adverse reactions. This makes it a promising therapeutic alternative or addition to standard metformin therapy [16,17].

Another natural product that is attracting increasing attention is berberine. Berberine is an isoquinoline alkaloid extracted from plants of the genus Berberis. Its mechanism of action involves the activation of AMP-activated protein kinase (AMPK), which is known to be a key regulator of cellular energy metabolism. Thus, berberine improves insulin sensitivity, reduces hepatic gluconeogenesis, affects the lipid profile, and contributes to the reduction of androgen production. Systematic reviews and meta-analyses show that in patients with PCOS and insulin resistance, berberine leads to a significant improvement in the indicators of carbohydrate and lipid metabolism. Some studies have reported metabolic effects of berberine comparable to those observed with metformin, including potentially favorable effects on selected lipid parameters [18,19].

The growing interest in myo-inositol and berberine reflects the current trend towards individualising treatment in PCOS. Given the metabolic heterogeneity of the syndrome, the choice of therapeutic strategy should be based not only on the reproductive manifestations of the disease but also on the individual metabolic risk, the degree of insulin resistance, and the tolerance to different therapeutic agents. Metformin remains the reference drug, but myo-inositol and berberine are used as dietary supplement- or nutraceutical-based approaches and have attracted interest as potential complementary strategies for the individualized management of women with PCOS. Their regulatory and guideline status differs from that of metformin, and the evidence supporting their clinical use remains less established.

Despite the increasing number of studies evaluating these interventions in women with PCOS, evidence regarding their comparative effects remains limited. Therefore, the present study aimed to describe six-month changes in anthropometric, metabolic, lipid, and androgen parameters among women with PCOS receiving myo-inositol, metformin, berberine, or combined metformin–berberine therapy in routine clinical practice, and to explore whether treatment-group differences persisted after adjustment for baseline characteristics. Given the observational design and non-random treatment allocation, the study was not designed to establish the superiority of one treatment over another.

## 2. Materials and Methods

### 2.1. Study Design

The current study was designed as a retrospective observational cohort study conducted at a specialized hospital for active treatment in obstetrics and gynecology with national coverage, located in Sofia, Bulgaria. The study evaluated six-month changes in anthropometric, metabolic, lipid, and androgen parameters among women receiving routinely prescribed insulin-sensitizing therapies in real-world clinical practice. Treatment allocation was non-random and reflected routine clinical decision-making by the treating physician. Therefore, the study was intended to describe changes within clinically selected treatment groups rather than to establish comparative treatment efficacy or superiority.

### 2.2. Study Population

Clinical records of women with PCOS treated at Ob/Gyn “Dr. Shterev Hospital”, Sofia, Bulgaria, between 1 October 2024 and 1 October 2025 were retrospectively reviewed. The analysis included women who initiated one of the studied therapeutic regimens and completed an uninterrupted six-month course, with clinical and laboratory data available both at baseline and after six months.

The inclusion criteria were: (1) diagnosis of PCOS according to the Rotterdam criteria; (2) no ongoing PCOS-specific pharmacological or supplement therapy at baseline; (3) initiation of treatment with one of the four evaluated regimens—myo-inositol, metformin, berberine, or combined metformin–berberine therapy; (4) completion of an uninterrupted six-month treatment course; and (5) availability of the required clinical and laboratory data at baseline and after six months.

The exclusion criteria were: (1) ongoing treatment with combined oral contraceptives, progestins, or other hormonal therapy that could influence metabolic or androgen parameters; (2) treatment with spironolactone; (3) concurrent fertility treatment; (4) discontinuation or change of the initially prescribed therapeutic regimen during the six-month follow-up; and (5) insufficient baseline or six-month clinical or laboratory data for the planned analyses. These criteria were applied to minimize the potential influence of concomitant therapies on the metabolic and androgen parameters evaluated during follow-up.

The assignment of treatment was not randomized and was based on standard clinical judgment by the treating physician instead of a predetermined trial procedure. Therapeutic choices were tailored based on the patient’s clinical and metabolic attributes, including body mass index (BMI) and the degree of insulin resistance, reproductive intentions, and therapeutic inclinations. Metformin was utilized in alignment with its recognized function in the treatment of metabolic characteristics of PCOS. Myo-inositol and berberine were employed as non-pharmacological supplement strategies in standard therapeutic practice. The 2023 International Evidence-based Guideline for PCOS indicates that inositol may be utilized based on personal choices and values, while recognizing the restricted clinical advantages and inconsistencies in the regulatory status and quality assurance of supplement formulations. Berberine is not explicitly endorsed as a treatment for PCOS in the existing international guidelines; hence, its application in this group represents personalized clinical practice rather than guideline-based pharmacological intervention.

Lifestyle interventions, including dietary intake and physical activity, were not standardized as part of the study and were not systematically documented or monitored in the retrospective dataset.

The participants were divided into four groups according to the therapy: myo-inositol (n = 38), administered at a dose of 2 × 1000 mg per day; metformin (n = 52), administered at a dose of 2 × 750 mg per day in the form of extended-release tablets; berberine (n = 28), administered at a dose of 2 × 500 mg per day; and combination therapy with metformin and berberine (n = 30), at the same dosage regimens. The following clinical and laboratory parameters were evaluated: anthropometric (body mass index, BMI), metabolic (fasting glucose, immunoreactive insulin and homeostasis model assessment of insulin resistance -HOMA-IR), lipid profile (total cholesterol, low-density lipoprotein cholesterol (LDL-C), high-density lipoprotein cholesterol (HDL-C) and triglycerides), as well as basal levels of hormonal indicators reflecting androgenic status (total testosterone, dehydroepiandrosterone sulfate- DHEAS, androstenedione and 17-hydroxyprogesterone). Baseline levels of testosterone, DHEAS, 17-OH-progesterone, follicule-stimulating hormone (FSH), luteinizing hormone (LH), prolactin, estradiol, and anti-Mullerian hormone (AMH) were measured in all patients between the third and the fifth days of spontaneous menstruation. All patients underwent a 75 g oral glucose tolerance test (OGTT), with plasma glucose and IRI measurements obtained at 0, 60, and 120 min. HOMA-IR index was calculated. The index of homeostasis model assessment of insulin resistance (HOMA-IR) was calculated as fasting serum insulin (μIU/mL) × fasting glucose (mmol/L)/22.5 [20,21,22].

### 2.3. Outcome Measures and Laboratory Results

Assessments were performed at baseline and after 6 months. The longitudinally evaluated outcomes included BMI; metabolic parameters comprising fasting plasma glucose, fasting insulin (IRI), and HOMA-IR; lipid parameters comprising total cholesterol, LDL-C, HDL-C, and triglycerides; and androgen-related hormonal parameters comprising total testosterone, androstenedione, DHEAS, and 17-OH-progesterone. Additional hormonal parameters, including AMH, FSH, LH, estradiol, prolactin, and thyroid-stimulating hormone (TSH), were assessed as part of the baseline clinical evaluation but were not included among the longitudinal treatment outcomes. Each laboratory variable was additionally classified as normal or abnormal against its reference range. Hormonal analysis includes testing of basal hormone levels during the early follicular phase (between the third and fifth day of the period), after at least 12 h of overnight fasting between 8:00 and 9:00 a.m. FSH, LH, Estradiol, Prolactin, TSH, testosterone, DHEAS, androstenedione, 17-OH-progesterone and AMH levels were determined using the Immunochemical analysis ECLIA (cobas 8000, Roche Diagnostics, Basel, Switzerland), with the laboratory’s reference interval being: FSH—3.5–12.5 mIU/L (early follicular phase); LH—2.4–12.6 mIU/L (early follicular phase), Estradiol—45.4–854 pmol/L (early follicular phase), Prolactin—125–625 mIU/L; TSH—0.3–4.20 mIU/L.; testosterone—0.22–2.9 nmol/L; DHEAS—1.3–13.3 µmol/L; androstenedione—4.29–13.68 nmol/L; 17-OH-progesterone—0.3–2.4 nmol/L (early follicular phase); and AMH—1–6.8 ng/mL. Immunoreactive insulin (IRI) levels were assessed using the electrochemiluminescence method (Roche Cobas 8000, Switzerland) with reference intervals for the respective laboratory of 2.6–24.9 mIU/L. The UV hexokinase method (IFCC) was used to assess plasma glucose levels during OGTT, with a reference range of 3.6–6.1 mmol/L. HOMA-IR was analyzed primarily as a continuous variable. For the descriptive categorical analysis, a HOMA-IR value of 2.5 was used as a pragmatic cutoff to classify participants according to insulin resistance status; this threshold was not considered a universal diagnostic criterion for insulin resistance [21]. Serum lipid parameters, including total cholesterol (TC), low-density lipoprotein cholesterol (LDL-C), high-density lipoprotein cholesterol (HDL-C), and triglycerides (TG), were measured in fasting venous blood samples obtained after an overnight fast of at least 8–12 h. The following laboratory reference ranges were used for interpretation of results: total cholesterol < 5.2 mmol/L, LDL-cholesterol < 3.0 mmol/L, HDL-cholesterol > 1.3 mmol/L, and triglycerides < 1.7 mmol/L. Values outside these ranges were considered abnormal and were used for the assessment of normalization rates during follow-up.

## 3. Data Preparation

For each paired variable, the change from baseline to 6 months (Δ = 6-month value—baseline value) was calculated, and each variable was categorized based on its reference range as normal or abnormal.

## 4. Statistical Analysis

Continuous variables were summarized as mean (SD) when approximately normally distributed and as median [Q1, Q3] when skewed; categorical variables were presented as frequencies and percentages. Normality was assessed using the Shapiro–Wilk test. Baseline differences among treatment groups were evaluated using one-way analysis of variance (ANOVA) for approximately normally distributed variables or the Kruskal–Wallis test for skewed variables.

Within-group changes from baseline to six months were assessed using the Wilcoxon signed-rank test. To account for multiple testing across the examined outcomes, *p*-values from the within-group analyses were adjusted separately within each treatment group using the Holm method. Both unadjusted and Holm-adjusted *p*-values are reported, with statistical inference based on the Holm-adjusted *p*-values.

Between-group differences in six-month change scores (Δ = 6-month value—baseline value) were evaluated using the Kruskal–Wallis test. When the overall Kruskal–Wallis test was statistically significant, post hoc pairwise comparisons were performed using Dunn’s test with Bonferroni adjustment for multiple comparisons.

Changes in the proportions of participants with abnormal values from baseline to six months were assessed using two-sided exact McNemar tests. To account for multiple testing across the examined categorical outcomes, *p*-values were adjusted separately within each treatment group using the Holm method. For the categorical analysis of androstenedione, only values above the upper reference limit were classified as abnormal, as this analysis was intended to reflect biochemical hyperandrogenism.

Given the substantial baseline differences among treatment groups, additional baseline-adjusted analyses were performed for all continuous outcomes evaluated longitudinally. For each outcome, the 6-month value was modeled as the dependent variable, with treatment group, the corresponding baseline value, age, and baseline BMI included as covariates. For BMI, the model included treatment group, baseline BMI, and age. Heteroscedasticity-consistent (HC3) standard errors were used to reduce sensitivity to heteroscedasticity. The overall treatment-group effect was assessed for each model. To account for multiple testing across the 12 adjusted outcome models, the resulting *p*-values for the overall treatment-group effects were corrected using the Holm method. Adjusted means with 95% confidence intervals (CIs) were estimated from these models. These adjusted analyses were considered supplementary because treatment allocation was non-randomized and residual confounding could not be excluded.

Associations among changes in study parameters (Δ = 6-month value—baseline value) were quantified using Spearman rank-order correlation coefficients (ρ). For selected correlations reported in the text, 95% CIs for Spearman’s ρ were estimated using bootstrap resampling. Squared Spearman coefficients (ρ^2^) were additionally reported as descriptive measures of association strength. Correlation strength was interpreted as very weak (|ρ| < 0.20), weak (0.20–0.39), moderate (0.40–0.59), strong (0.60–0.79), and very strong (≥0.80).

All statistical tests were two-tailed, with α = 0.05. HOMA-IR was analyzed as a continuous variable in the primary analyses; the cutoff of 2.5 was used only for supplementary descriptive categorical analyses. No imputation of missing data was performed, and analyses were based on available paired observations for each variable. Statistical analyses were performed using IBM SPSS Statistics, Version 23. The additional baseline-adjusted analyses were performed using Python version 3.13.5 and the statsmodels package version 0.14.6.

## 5. Results

Baseline characteristics of the study population are presented in Table 1. The 4 treatment groups were similar with respect to demographic and hormonal characteristics at baseline. Significant differences between treatment groups were observed for BMI (*p* < 0.001), fasting plasma glucose (*p* < 0.001), IRI (*p* < 0.001), HOMA-IR (*p* < 0.001), total cholesterol (*p* = 0.04), HDL-C (*p* = 0.02), and triglycerides (*p* = 0.02). The Metformin group exhibited the highest BMI, fasting plasma glucose (FPG), IRI, and HOMA-IR values.

Intra-group evaluations revealed alterations in many anthropometric, metabolic, lipid, and androgen metrics following a six-month treatment period (Table 2). Following the use of the Holm method for multiple testing adjustment within each treatment cohort, several modifications continued to exhibit statistical significance. In the metformin cohort, decreases in BMI, FPG, IRI, HOMA-IR, total cholesterol, LDL-C, triglycerides, testosterone, androstenedione, DHEAS, and 17-OH-progesterone, and an elevation in HDL-C, persisted statistically significant post-Holm adjustment (all adjusted *p* < 0.001). In the myo-inositol cohort, statistically significant decreases were observed in IRI (adjusted *p* = 0.002), HOMA-IR (adjusted *p* = 0.009), total cholesterol (adjusted *p* = 0.042), LDL-C (adjusted *p* = 0.011), triglycerides (adjusted *p* = 0.042), androstenedione (adjusted *p* < 0.001), and DHEAS (adjusted *p* < 0.001). The changes in BMI, FPG, HDL-C, testosterone, and 17-OH-progesterone lost their statistical significance following correction. In the berberine cohort, substantial decreases in BMI (adjusted *p* < 0.001), IRI (adjusted *p* < 0.001), HOMA-IR (adjusted *p* < 0.001), total cholesterol (adjusted *p* = 0.006), triglycerides (adjusted *p* < 0.001), and androstenedione (adjusted *p* = 0.028) persisted following Holm adjustment. Alterations in FPG, LDL-C, HDL-C, testosterone, DHEAS, and 17-OH-progesterone lost their statistical significance following correction. In the cohort receiving both metformin and berberine, significant decreases in BMI, IRI, HOMA-IR, total cholesterol, LDL-C, triglycerides, testosterone, androstenedione, DHEAS, and 17-OH-progesterone, alongside a notable increase in HDL-C, persisted after Holm adjustment (all adjusted *p* < 0.05). FPG did not exhibit a statistically meaningful alteration.

The categorical evaluation utilizing established laboratory reference ranges is illustrated in Table 3. Following the application of the Holm method for multiple testing correction, only a few alterations in the proportions of participants exhibiting abnormal values retained statistical significance. In the metformin cohort, notable decreases were noted in the percentages of individuals exhibiting abnormal BMI (88.5% to 71.2%; Holm-adjusted *p* = 0.031), FPG (21.2% to 0%; adjusted *p* = 0.009), IRI (42.3% to 1.9%; adjusted *p* < 0.001), total cholesterol (40.4% to 7.7%; adjusted *p* < 0.001), and triglycerides (27.5% to 2.0%; adjusted *p* = 0.002). Modifications in the other category outcomes lost their statistical significance following correction. In the myo-inositol cohort, the percentage of individuals exhibiting abnormal LDL-C levels fell from 34.2% to 5.3%, maintaining statistical significance post-Holm adjustment (adjusted *p* = 0.041). No other categorical result persisted as statistically significant following correction. In the berberine cohort, the percentage of individuals exhibiting abnormal triglyceride levels diminished from 39.3% at baseline to 0% at the six-month mark (adjusted *p* = 0.012). No additional category outcomes retained statistical significance following the Holm correction. In the cohort receiving both metformin and berberine, the percentage of individuals exhibiting abnormal LDL-C levels fell from 43.3% to 10.0% (adjusted *p* = 0.023). No other categorical result persisted as statistically significant following correction. For androstenedione, solely values exceeding the upper reference threshold were deemed abnormal in the evaluation of biochemical hyperandrogenism. The percentage of subjects exhibiting increased androstenedione levels was minimal at baseline, and no statistically significant categorical alterations were detected in any treatment cohort following adjustment for multiple comparisons. These categorical findings should be distinguished from the analysis of continuous androstenedione concentrations in Table 2, as the two analyses address different aspects of the data.

Between-group comparisons of the six-month changes in anthropometric, metabolic, lipid, and androgen-related parameters are presented in Table 4.

Due to the significant baseline differences between treatment groups, supplementary baseline-adjusted analyses were conducted for all continuous outcomes assessed over time. For every result, the 6-month value was conceptualized as the dependent variable, incorporating treatment group, the relevant baseline value, age, and baseline BMI as covariates. The model for BMI incorporated the treatment group, initial BMI, and age, without further adjustment for initial BMI as an independent covariate. Robust standard errors were implemented to mitigate sensitivity to heteroscedasticity. The overall impact of the treatment group was evaluated for each model. To address the issue of multiple testing across the 12 modified outcome models, the *p*-values for the total treatment-group effects were adjusted utilizing the Holm method. The modified analyses were deemed supplemental due to the non-randomized nature of treatment allocation and the inability to rule out residual confounding.

Due to the significant initial differences among treatment groups, baseline-adjusted analyses were conducted for all continuous outcomes assessed over the entire period (Table 5). Upon adjusting for the relevant baseline value, age, and initial BMI, along with the Holm correction applied across the 12 outcome models, no statistically significant overall effects of the treatment groups were detected for BMI, FPG, IRI, HOMA-IR, total cholesterol, LDL-C, HDL-C, triglycerides, testosterone, or androstenedione (all Holm-adjusted *p* > 0.05). Overall, treatment-group effects for DHEAS (Holm-adjusted *p* < 0.001) and 17-OH-progesterone (Holm-adjusted *p* = 0.010) were statistically significant. These comprehensive results reveal variations among the treatment cohorts in modified 6-month metrics, although they do not, on their own, specify which particular treatment groups exhibited changes. Due to the observational nature of the non-randomized treatment assignment and the potential for residual confounding, these results must be approached with caution and should not be regarded as proof of comparative treatment superiority.

Spearman correlations among the 6-month change scores (Δ = follow-up—baseline) are presented separately for each treatment group in Table 6, Table 7, Table 8 and Table 9.

The strongest and most consistent association across the treatment groups was observed between changes in fasting insulin and HOMA-IR: metformin, ρ = 0.879 (95% CI 0.712–0.966; ρ^2^ = 0.773; *p* < 0.001); myo-inositol, ρ = 0.887 (95% CI 0.732–0.966; ρ^2^ = 0.786; *p* < 0.001); berberine, ρ = 0.684 (95% CI 0.344–0.887; ρ^2^ = 0.467; *p* < 0.001); and metformin + berberine, ρ = 0.879 (95% CI 0.741–0.946; ρ^2^ = 0.772; *p* < 0.001). In the metformin and myo-inositol groups, reductions in BMI showed weak-to-moderate positive correlations with reductions in IRI (metformin: ρ = 0.38, *p* = 0.005; myo-inositol: ρ = 0.56, *p* < 0.001) and HOMA-IR (metformin: ρ = 0.31, *p* = 0.024; myo-inositol: ρ = 0.44, *p* = 0.005). In the myo-inositol group, reductions in BMI also showed weak correlations with reductions in LDL-C (ρ = 0.36, *p* = 0.025) and increases in HDL-C (ρ = −0.34, *p* = 0.036).

Changes in FPG demonstrated moderate positive correlations with changes in HOMA-IR in the metformin (ρ = 0.42, *p* = 0.002), myo-inositol (ρ = 0.55, *p* < 0.001), and metformin + berberine groups (ρ = 0.54, *p* = 0.002). In the berberine group, FPG showed strong positive correlations with HOMA-IR (ρ = 0.69, *p* < 0.001) and IRI (ρ = 0.68, *p* < 0.001).

In the metformin group, testosterone showed a weak positive correlation with DHEAS (ρ = 0.28, *p* = 0.043). In the myo-inositol group, testosterone demonstrated a weak positive correlation with 17-OH-progesterone (ρ = 0.33, *p* = 0.046). In the metformin + berberine group, IRI showed a moderate positive correlation with DHEAS (ρ = 0.48, *p* = 0.007). In the berberine group, testosterone showed weak negative correlations with HOMA-IR (ρ = −0.42, *p* = 0.026) and total cholesterol (ρ = −0.41, *p* = 0.032), while androstenedione demonstrated a moderate negative correlation with HDL-C (ρ = −0.50, *p* = 0.007).

Overall, significant correlations were predominantly observed among markers of insulin resistance and lipid metabolism and ranged from weak to very strong, whereas associations among androgen parameters were generally weak and less consistent across treatment groups.

Within-group correlation matrices (6-month change).

The corresponding correlations between changes over six months in the myo-inositol and berberine groups are presented in Table 7 and Table 8, respectively.

The correlations among six-month changes in the combined metformin–berberine group are presented in Table 9.

**Figure 1:**
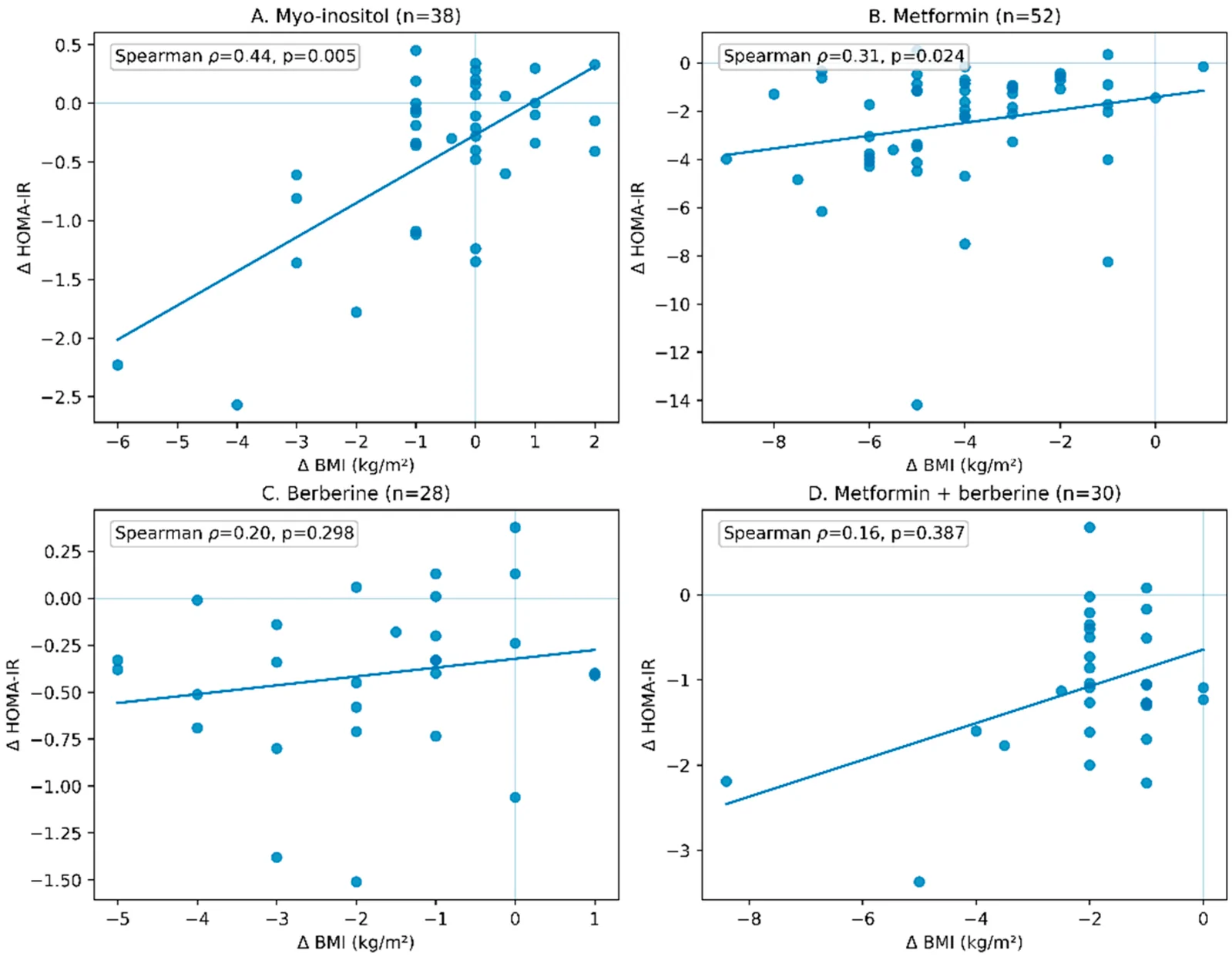
Association between 6-month changes in BMI and HOMA-IR by treatment group.

**Figure 2:**
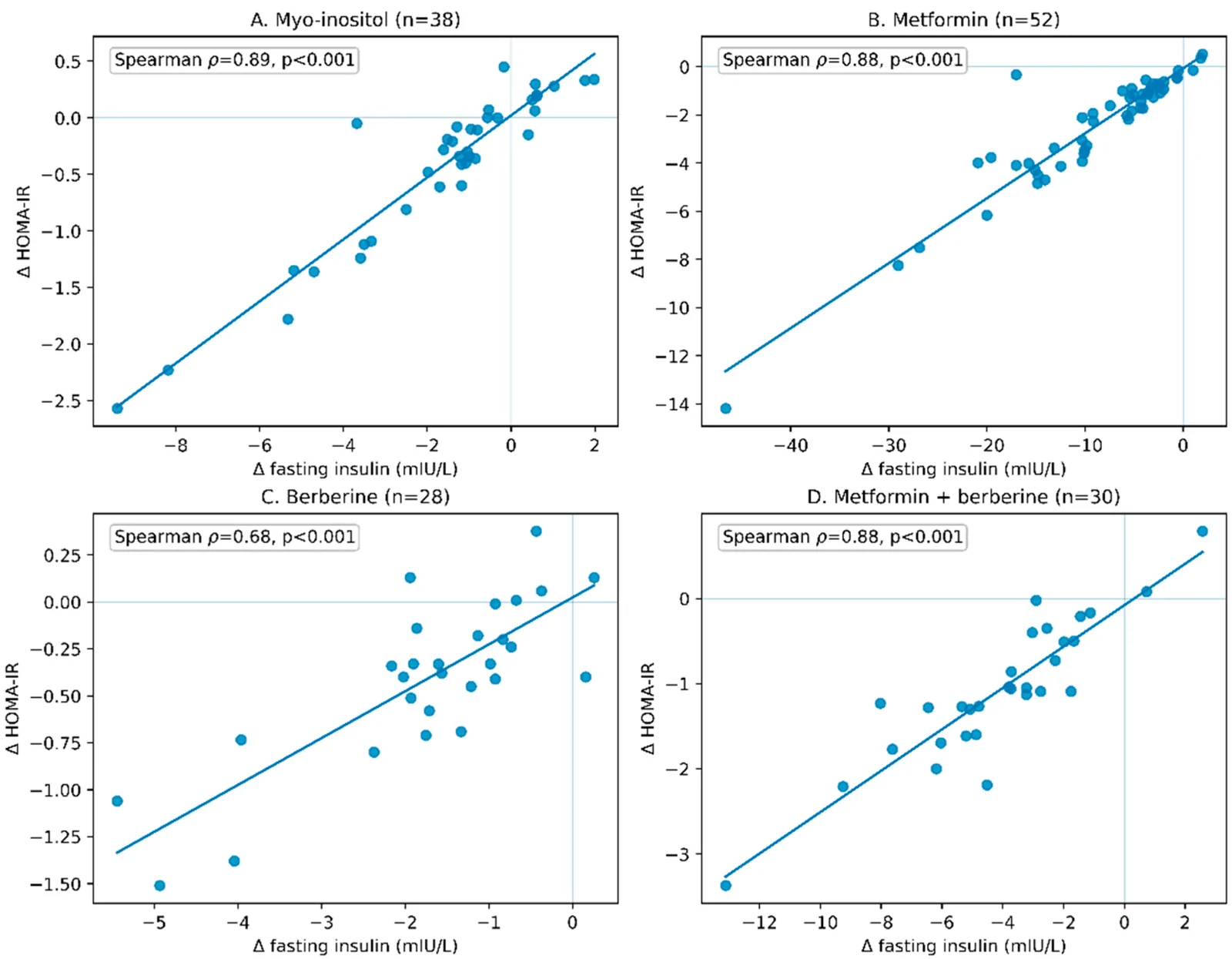
Association between 6-month changes in fasting insulin and Homa-IR by treatment group.

Selected correlations are additionally presented graphically in Figure 1 and Figure 2. Figure 1 illustrates the associations between changes in BMI and HOMA-IR across the four treatment groups, whereas Figure 2 presents the consistently positive associations between changes in fasting insulin and HOMA-IR. The latter should be interpreted in the context of HOMA-IR being a derived index calculated from fasting insulin and fasting plasma glucose.

## 6. Discussion

Our retrospective observational cohort study aimed to identify six-month alterations in anthropometric, metabolic, lipid, and androgen metrics among women with PCOS undergoing myo-inositol, metformin, berberine, or a combination of metformin and berberine treatment in standard clinical settings. Numerous intra-group alterations retained statistical significance after multiple testing correction; still, the treatment cohorts exhibited considerable disparities in their initial metabolic traits, especially for BMI, fasting plasma glucose, fasting insulin, and HOMA-IR. In the baseline-adjusted evaluations, no statistically significant overall effects of the treatment groups persisted for the primary metabolic outcomes following Holm adjustment. Notable overall effects of the treatment groups were detected for DHEAS and 17-OH-progesterone; nevertheless, these general results do not pinpoint specific differences between treatment pairs and should be interpreted with caution due to the non-randomized treatment assignment and possible residual confounding. Thus, the extent of the detected intra-group alterations must not be construed as proof of comparative treatment efficacy. The results define trends in metabolic and hormonal alterations among clinically chosen patient cohorts receiving treatment in standard real-world settings.

A notable advantage of the current research is the assessment of several metabolically oriented therapeutic strategies in standard clinical settings. In the intra-group evaluations, metformin demonstrated statistically significant changes in various anthropometric, glycemic, lipid, and androgen metrics following adjustments for multiple comparisons. Notable intra-group alterations were also detected with the combination of metformin and berberine therapy, in addition to myo-inositol and berberine monotherapy for particular effects. These results are scientifically credible considering the various routes via which these interventions could affect insulin sensitivity and metabolic control. Metformin predominantly diminishes hepatic glucose synthesis and enhances insulin sensitivity, while berberine is noted for its effects on AMP-activated protein kinase signaling, lipid metabolism, and cellular energy regulation. Nonetheless, the current observational evidence does not demonstrate that the amalgamation of metformin and berberine yields superior metabolic effectiveness compared to either treatment individually.

The categorical examination of values beyond the established reference ranges offered an additional viewpoint on the continuous outcome evaluations. Following adjustments for multiple comparisons, the decrease in the percentage of participants exhibiting aberrant values remained statistically significant for several parameters within the metformin cohort, while a lesser number of category alterations retained significance in the alternative therapy groups. These results must be understood considering the significant initial disparities among the groups. Women administered metformin exhibited a less advantageous initial metabolic profile, thus possessing a heightened capacity for progression toward the reference range throughout subsequent evaluations. Thus, variations in normalization patterns might indicate, at least in part, the initial metabolic severity and the choice of therapeutic treatment rather than disparities in the effectiveness of treatments.

Individuals with more significant metabolic irregularities were more inclined to be prescribed metformin-inclusive treatment plans in standard clinical settings. This trend aligns with therapy choices determined by initial metabolic severity and should not be construed as proof that individuals with heightened metabolic dysfunction experience greater relative advantages from these medications. Despite the observation of more substantial absolute within-group changes for various outcomes in the metformin groups, the baseline-adjusted analyses failed to reveal statistically significant treatment-group effects for the primary metabolic outcomes following adjustments for multiple comparisons. The impact of baseline metabolic severity on treatment response remains ambiguous and necessitates assessment in future comparative research. This approach to personalized treatment selection aligns with the 2023 International Evidence-based Guideline for the Evaluation and Management of PCOS, which underscores the importance of metabolic risk, clinical attributes, and unique patient factors in determining management strategies. This guideline emphasizes the importance of interventions not only for reproductive outcomes but also for long-term cardiometabolic health [4].

The differences observed among the treatment groups raise the possibility that baseline metabolic characteristics may influence treatment response. However, the present observational study was not designed to identify which patients are most likely to benefit from a specific therapeutic approach. Prospective randomized studies, including analyses stratified by baseline metabolic characteristics, are needed to address this question.

The observed within-group changes are broadly consistent with previous studies evaluating metformin and berberine in women with PCOS. Meta-analyses evaluating metformin have reported improvements in insulin sensitivity and serum insulin concentrations, together with effects on reproductive outcomes [23,24]. In the present cohort, statistically significant within-group reductions in BMI, fasting plasma glucose, fasting insulin, and HOMA-IR remained in the metformin group after correction for multiple testing. However, the metformin group also had a substantially less favorable metabolic profile at baseline, and the baseline-adjusted analyses did not demonstrate a significant overall treatment-group effect for these principal metabolic outcomes. Therefore, the magnitude of the observed changes should not be interpreted as evidence that metformin was more effective than the other evaluated interventions. The findings observed in the berberine group are also broadly consistent with published evidence suggesting potential effects on insulin resistance and lipid metabolism [17,18]. In our cohort, within-group reductions in BMI, fasting insulin, HOMA-IR, total cholesterol, triglycerides, and androstenedione remained statistically significant after Holm correction. Nevertheless, direct comparison of the magnitude of these changes with those observed in the other treatment groups is limited by the non-randomized treatment allocation and baseline differences between groups.

The findings for combined metformin–berberine therapy are also of interest. Within this group, statistically significant changes remained after Holm correction for BMI, fasting insulin, HOMA-IR, total cholesterol, LDL-C, HDL-C, triglycerides, testosterone, androstenedione, DHEAS, and 17-OH-progesterone, whereas the change in fasting plasma glucose was not statistically significant. Although the complementary biological mechanisms of metformin and berberine provide a rationale for investigating their combined use, the present study was not designed to establish pharmacological synergy or additive treatment effects. Moreover, the baseline-adjusted analyses did not demonstrate a statistically significant overall treatment-group effect for the principal metabolic outcomes. Therefore, the observed within-group changes with combination therapy should be considered hypothesis-generating and require confirmation in prospective controlled studies.

The results of myo-inositol must also be understood within the framework of the initial attributes of this therapy cohort. Following Holm correction, notable within-group decreases persisted for fasting insulin, HOMA-IR, total cholesterol, LDL-C, triglycerides, androstenedione, and DHEAS, while alterations in BMI, fasting plasma glucose, HDL-C, testosterone, and 17-OH-progesterone were not statistically significant. These results align partially with earlier research indicating possible influences of myo-inositol on insulin sensitivity and certain metabolic and reproductive results in women diagnosed with PCOS [16,17]. The relatively advantageous initial metabolic characteristics of the myo-inositol cohort, such as reduced BMI, fasting glucose, and HOMA-IR levels, might have constrained the extent of detectable change during the follow-up period. Nonetheless, the current research does not permit determinations on whether initial metabolic severity influences the comparative response to myo-inositol or the other assessed treatments.

The hormonal results necessitate individual evaluation. Following adjustments for multiple comparisons in the intra-group evaluations, androstenedione levels exhibited a notable decline across all four treatment cohorts, while substantial decreases in testosterone were observed solely in the metformin and the combined metformin–berberine groups. DHEAS exhibited a notable reduction in the metformin, myo-inositol, and combined-treatment cohorts, whereas 17-OH-progesterone showed a substantial decline in the metformin and combined-treatment cohorts. In the baseline-adjusted evaluations, notable overall treatment-group effects persisted for DHEAS and 17-OH-progesterone following Holm adjustment. These collective effects suggest variations in adjusted six-month concentrations between the treatment groups, although they do not determine whether intervention yielded a more significant treatment impact.

Biochemical hyperandrogenism is associated with insulin resistance and hyperinsulinemia that are known stimulators of theca cells in the ovary to produce androgens and inhibitors of production of sex hormone binding globulin (SHBG) in the liver [12,14,25,26].

The observed hormonal changes are broadly consistent with previous studies evaluating these interventions in women with PCOS. Previous evidence has reported reductions in circulating androgen concentrations during metformin treatment, together with improvements in insulin sensitivity and metabolic parameters [23,24]. Myo-inositol has also been investigated for its potential effects on insulin signaling and endocrine parameters, including circulating androgen concentrations and ovulatory function [16,17]. Evidence regarding the hormonal effects of berberine in PCOS remains more limited, although previous studies have reported changes in both androgen and metabolic parameters following berberine administration [18,19]. Nevertheless, differences between the treatment groups in the present study should not be interpreted as evidence of comparative hormonal efficacy because of the observational design, non-randomized treatment allocation, and baseline differences among groups. The correlation analyses provide additional descriptive information regarding the relationships among changes in metabolic parameters during follow-up.

The correlation analyses provide additional descriptive information regarding the relationships among changes in metabolic parameters during follow-up. Strong positive correlations between changes in fasting insulin and HOMA-IR were observed across all four treatment groups. However, these associations should be interpreted cautiously because fasting insulin is a component of the HOMA-IR calculation. Positive correlations between changes in BMI and indices of insulin resistance were also observed in the metformin and myo-inositol groups, suggesting that changes in body weight and metabolic parameters occurred in parallel. Although these findings are consistent with established pathophysiological models linking insulin resistance, obesity, and hyperandrogenism in PCOS, they do not establish a causal relationship or identify the mechanisms underlying the observed treatment responses [12,14,25,26].

The clinical relevance of these findings should be interpreted cautiously. Although statistically significant changes were observed in several metabolic and hormonal parameters, statistical significance does not necessarily translate into a clinically meaningful reduction in long-term cardiometabolic risk. Moreover, the present study was neither designed nor powered to assess incident type 2 diabetes, cardiovascular events, or other hard clinical outcomes, and the six-month follow-up period was insufficient to determine whether the observed metabolic and biochemical changes translate into sustained long-term clinical benefit.

## 7. Limitations and Strengths

This study has several limitations.

The observational and non-randomized design does not allow causal conclusions regarding the comparative effects of the studied interventions. Treatment allocation reflected routine clinical decision-making rather than random assignment, and substantial baseline differences were present among the treatment groups. In particular, women receiving metformin had higher baseline BMI, fasting plasma glucose, fasting insulin, and HOMA-IR values than participants in the other groups. These differences are consistent with potential confounding by indication, as patients with more pronounced metabolic abnormalities may have been more likely to receive metformin-containing treatment. Regression to the mean may also have contributed to the larger absolute changes observed in groups with more abnormal baseline values. Although baseline-adjusted analyses were performed to partially address these differences, residual confounding cannot be excluded. Consequently, differences in the magnitude of change between groups should not be interpreted as evidence of comparative treatment efficacy or superiority.

Additional limitations include the relatively small sample sizes of some treatment groups, which may have reduced statistical power, particularly for hormonal outcomes. The six-month follow-up allowed assessment of short-term metabolic and endocrine changes but not their long-term persistence or their relationship with cardiovascular, reproductive, or diabetes-related clinical outcomes. Because the analysis included patients who completed an uninterrupted six-month treatment course, treatment-specific discontinuation rates and reasons for discontinuation could not be reliably reconstructed from the retrospective dataset. Adherence and adverse events were not systematically documented and therefore could not be reliably evaluated. Other potentially relevant parameters, including SHBG, free androgen index (FAI), and body composition, were also not systematically assessed. Finally, lifestyle interventions, including dietary intake and physical activity, were neither standardized nor systematically monitored. Their contribution to the observed anthropometric and metabolic changes therefore cannot be separated from changes occurring during pharmacological or supplement-based treatment.

Despite these limitations, the study has several strengths. It reflects routine clinical practice and provides longitudinal real-world data on anthropometric, metabolic, lipid, and androgen parameters in women with PCOS receiving four different therapeutic approaches. The availability of paired baseline and six-month measurements allowed evaluation of within-group changes across a broad range of metabolic and hormonal outcomes. In addition, correction for multiple testing and supplementary baseline-adjusted analyses were applied to improve the robustness and transparency of the statistical interpretation. The study therefore provides hypothesis-generating real-world evidence that may inform the design of future prospective comparative studies.

## 8. Conclusions

The present six-month retrospective observational study identified changes in anthropometric, metabolic, lipid, and androgen parameters among women with PCOS receiving metformin, myo-inositol, berberine, or combined metformin–berberine therapy in routine clinical practice. After correction for multiple testing, several within-group changes remained statistically significant across the evaluated treatment groups. However, the groups differed substantially in their baseline metabolic characteristics, reflecting non-random treatment selection in routine clinical practice.

In the baseline-adjusted analyses, no statistically significant overall treatment-group effects remained for the principal metabolic outcomes after correction for multiple testing, whereas significant overall treatment-group effects were observed for DHEAS and 17-OH-progesterone. These findings should not be interpreted as evidence of comparative treatment efficacy or superiority because of the observational design, non-randomized treatment allocation, and potential residual confounding. Rather, the study provides real-world observational data on metabolic and hormonal changes occurring within clinically selected treatment groups. Prospective controlled studies with larger sample sizes and longer follow-up are needed to determine the comparative effectiveness of these therapeutic approaches and to investigate patient characteristics that may be associated with treatment response.

## Figures and Tables

**Table 1:** Baseline characteristics by treatment group.

	Group				
Characteristic	Myo-Inositol (n = 38)	Berberine (n = 28)	Metformin (n = 52)	Metformin + Berberine (n = 30)	*p*
Age (years)	28.5 [24.0, 32.0]	30.0 [25.8, 33.2]	30.0 [25.0, 33.0]	28.0 [25.0, 31.0]	0.66
BMI (kg/m^2^)	20.0 [19.0, 25.8]	25.0 [22.0, 28.0]	33.0 [27.8, 35.0]	25.0 [24.0, 28.8]	<0.001
AMH (ng/mL)	7.2 [6.2, 10.1]	8.5 [6.3, 10.0]	7.3 [6.1, 9.7]	7.9 [5.8, 8.9]	0.70
LH (IU/L)	8.9 [7.2, 11.8]	10.2 [8.5, 12.0]	9.0 [7.3, 11.9]	10.6 [8.5, 13.6]	0.36
FSH (IU/L)	5.4 [4.7, 6.3]	5.7 [5.2, 6.2]	5.5 [4.7, 6.6]	6.4 [5.6, 8.2]	0.08
Estradiol	123.0 [89.0, 171.7]	124.5 [74.0, 154.6]	143.0 [94.5, 194.4]	153.0 [113.0, 180.8]	0.26
Prolactin (mIU/L)	486.5 [371.5, 687.8]	401.0 [265.8, 634.2]	657.0 [328.5, 743.8]	503.5 [244.2, 654.5]	0.15
TSH	2.30 [1.55, 3.35]	1.77 [1.41, 3.04]	2.56 [1.70, 3.40]	2.77 [1.58, 3.90]	0.22
Testosterone (nmol/L)	1.83 [1.13, 2.15]	1.62 [1.21, 2.15]	1.44 [1.08, 1.90]	1.61 [1.23, 2.07]	0.35
DHEAS (µmol/L)	8.9 [7.0, 12.3]	10.3 [9.0, 12.6]	8.7 [6.9, 10.3]	9.0 [6.0, 10.2]	0.24
Androstenedione (nmol/L)	6.2 [4.9, 8.5]	5.4 [3.6, 7.9]	6.4 [4.1, 8.6]	5.6 [4.1, 8.4]	0.62
17-OH-progesterone (nmol/L)	2.71 [1.34, 3.47]	2.25 [1.31, 3.67]	2.15 [1.44, 2.72]	2.26 [1.48, 3.48]	0.80
FPG (mmol/L)	5.1 (0.5)	5.2 (0.5)	5.6 (0.5)	5.3 (0.6)	<0.001
IRI (mIU/L)	8.2 [7.3, 13.1]	13.1 [10.2, 14.6]	22.7 [16.3, 32.5]	18.2 [13.3, 20.2]	<0.001
HOMA-IR	1.8 [1.6, 3.2]	2.9 [2.5, 3.2]	5.5 [4.1, 8.3]	3.8 [2.9, 4.9]	<0.001
Total cholesterol (mmol/L)	4.8 (0.5)	4.8 (0.5)	5.1 (0.6)	4.9 (0.5)	0.04
LDL-C (mmol/L)	2.70 [2.42, 3.00]	3.05 [2.10, 3.65]	2.70 [2.48, 3.77]	2.90 [2.50, 3.54]	0.55
HDL-C (mmol/L)	1.41 (0.20)	1.35 (0.17)	1.28 (0.20)	1.31 (0.14)	0.02
Triglycerides (mmol/L)	1.45 [1.24, 1.65]	1.63 [1.53, 1.71]	1.56 [1.44, 1.70]	1.46 [1.23, 1.62]	0.02

Note. Values are M (SD) for symmetric variables and Mdn [Q1, Q3] for skewed variables. Between-group baseline comparison by one-way ANOVA (symmetric) or Kruskal–Wallis test (skewed). Abbreviations: BMI, body mass index; AMH, anti-Müllerian hormone; LH, luteinizing hormone; FSH, follicle-stimulating hormone; TSH, thyroid-stimulating hormone; DHEAS, dehydroepiandrosterone sulfate; 17-OH-progesterone, 17-hydroxyprogesterone; FPG, fasting plasma glucose; IRI, immunoreactive insulin; HOMA-IR, homeostasis model assessment of insulin resistance; LDL-C, low-density lipoprotein cholesterol; HDL-C, high-density lipoprotein cholesterol.

**Table 2:** Within-group changes from baseline to 6 months, by treatment group.

Outcome	Metformin Baseline	6 Months	*p*-Value	Holm-Adjusted *p*-Value	Myo-Inositol Baseline	6 Months	*p*-Value	Holm-Adjusted *p*-Value	Berberine Baseline	6 Months	*p*-Value	Holm-Adjusted *p*-Value	Metformin + Berberine Baseline	6 Months	*p*-Value	Holm-Adjusted *p*-Value
BMI (kg/m^2^)	33.0 [27.8, 35.0]	28.0 [24.0, 30.0]	<0.001	**<0.001**	20.0 [19.0, 25.8]	20.0 [19.0, 23.3]	0.070	0.210	25.0 [22.0, 28.0]	22.5 [21.0, 26.3]	<0.001	**<0.001**	25.0 [24.0, 28.8]	23.5 [22.0, 27.0]	<0.001	**<0.001**
FPG (mmol/L)	5.65 [5.22, 6.01]	5.21 [5.06, 5.46]	<0.001	**<0.001**	5.20 [4.82, 5.50]	5.07 [4.87, 5.20]	0.226	0.317	5.20 [5.00, 5.43]	5.14 [5.05, 5.33]	0.649	1.000	5.27 [5.05, 5.62]	5.16 [4.99, 5.30]	0.128	0.128
IRI (mIU/L)	22.70 [16.34, 32.50]	15.92 [13.09, 19.70]	<0.001	**<0.001**	8.18 [7.29, 13.08]	7.93 [6.92, 10.79]	<0.001	**0.002**	13.05 [10.23, 14.62]	11.19 [9.53, 12.06]	<0.001	**<0.001**	18.17 [13.29, 20.20]	11.88 [9.93, 15.36]	<0.001	**<0.001**
HOMA-IR	5.5 [4.1, 8.3]	3.8 [3.0, 4.5]	<0.001	**<0.001**	1.8 [1.6, 3.2]	1.8 [1.5, 2.4]	0.001	**0.009**	2.9 [2.5, 3.2]	2.5 [2.2, 2.7]	<0.001	**<0.001**	3.8 [2.9, 4.9]	2.7 [2.1, 3.5]	<0.001	**<0.001**
Total cholesterol (mmol/L)	5.03 [4.57, 5.45]	4.54 [4.23, 4.90]	<0.001	**<0.001**	4.88 [4.50, 5.11]	4.56 [4.23, 4.77]	0.006	**0.042**	4.71 [4.30, 5.11]	4.37 [4.13, 4.67]	<0.001	**0.006**	5.01 [4.55, 5.17]	4.44 [4.23, 4.67]	<0.001	**0.003**
LDL-C (mmol/L)	2.70 [2.48, 3.77]	2.25 [2.04, 2.90]	<0.001	**<0.001**	2.70 [2.42, 3.00]	2.39 [2.12, 2.71]	0.001	**0.011**	3.05 [2.10, 3.65]	2.60 [2.10, 3.12]	0.013	0.081	2.90 [2.50, 3.54]	2.26 [2.00, 2.60]	<0.001	**<0.001**
HDL-C (mmol/L)	1.28 [1.15, 1.42]	1.40 [1.29, 1.50]	<0.001	**<0.001**	1.45 [1.23, 1.55]	1.46 [1.31, 1.59]	0.158	0.317	1.40 [1.23, 1.46]	1.40 [1.33, 1.48]	0.040	0.161	1.31 [1.23, 1.44]	1.44 [1.30, 1.55]	0.002	**0.009**
Triglycerides (mmol/L)	1.57 [1.45, 1.70]	1.24 [1.12, 1.41]	<0.001	**<0.001**	1.45 [1.24, 1.65]	1.33 [1.21, 1.49]	0.006	**0.042**	1.63 [1.53, 1.71]	1.40 [1.30, 1.51]	<0.001	**<0.001**	1.46 [1.23, 1.62]	1.34 [1.23, 1.52]	0.011	**0.044**
Testosterone (nmol/L)	1.44 [1.08, 1.90]	1.11 [0.89, 1.54]	<0.001	**<0.001**	1.83 [1.13, 2.15]	1.66 [1.25, 1.90]	0.021	0.104	1.62 [1.21, 2.15]	1.45 [1.22, 1.92]	0.031	0.157	1.61 [1.23, 2.07]	1.46 [1.20, 1.73]	0.013	**0.044**
Androstenedione (nmol/L)	6.38 [4.07, 8.61]	4.23 [3.20, 7.15]	<0.001	**<0.001**	6.23 [4.95, 8.46]	5.08 [4.10, 7.46]	<0.001	**<0.001**	5.43 [3.63, 7.88]	4.37 [3.46, 6.57]	0.004	**0.028**	5.62 [4.14, 8.41]	4.28 [3.26, 7.50]	<0.001	**<0.001**
DHEAS (µmol/L)	8.69 [6.90, 10.33]	6.75 [5.04, 8.88]	<0.001	**<0.001**	8.94 [7.03, 12.30]	8.52 [6.22, 10.28]	<0.001	**<0.001**	10.33 [8.98, 12.60]	9.87 [7.98, 11.12]	0.101	0.303	9.03 [6.02, 10.23]	8.50 [5.14, 9.87]	0.002	**0.009**
17-OH-progesterone (nmol/L)	2.15 [1.44, 2.72]	1.94 [1.29, 2.37]	<0.001	**<0.001**	2.71 [1.34, 3.47]	2.12 [1.69, 2.89]	0.026	0.104	2.25 [1.31, 3.67]	2.28 [1.69, 3.17]	0.569	1.000	2.26 [1.48, 3.48]	1.98 [1.54, 2.88]	0.023	**0.046**

Abbreviations: BMI, body mass index; FPG, fasting plasma glucose; IRI, immunoreactive insulin; HOMA-IR, homeostasis model assessment of insulin resistance; TC, total cholesterol; LDL-C, low-density lipoprotein cholesterol; HDL-C, high-density lipoprotein cholesterol; TG, triglycerides; DHEAS, dehydroepiandrosterone sulfate; 17-OH-progesterone, 17-hydroxyprogesterone. Note: Values are presented as median [Q1, Q3]. Within-group changes from baseline to 6 months were assessed using the Wilcoxon signed-rank test. To account for multiple testing across the examined outcomes, *p*-values were adjusted separately within each treatment group using the Holm method. Both unadjusted and Holm-adjusted *p*-values are presented; statistical inference was based on the Holm-adjusted *p*-values. Bold values indicate Holm-adjusted *p* < 0.05.

**Table 3:** Proportion of participants with abnormal values at baseline and after 6 months, by treatment group.

Parameter	Metformin Baseline n (%)	6 Months n (%)	*p*	Holm p	Myo-Inositol Baseline n (%)	6 Months n (%)	*p*	Holm p	Berberine Baseline n (%)	6 Months n (%)	*p*	Holm p	Metformin + Berberine Baseline n (%)	6 Months n (%)	*p*	Holm p
BMI	46 (88.5)	37 (71.2)	0.004	**0.031**	10 (26.3)	9 (23.7)	1.000	1.000	15 (53.6)	11 (39.3)	0.125	0.875	17 (56.7)	11 (36.7)	0.031	0.344
FPG	11 (21.2)	0 (0.0)	<0.001	**0.009**	0 (0.0)	0 (0.0)	1.000	1.000	0 (0.0)	0 (0.0)	1.000	1.000	3 (10.0)	0 (0.0)	0.250	1.000
IRI	22 (42.3)	1 (1.9)	<0.001	**<0.001**	1 (2.6)	0 (0.0)	1.000	1.000	0 (0.0)	0 (0.0)	1.000	1.000	2 (6.7)	0 (0.0)	0.500	1.000
HOMA-IR	49 (94.2)	45 (86.5)	0.125	0.500	13 (34.2)	8 (21.1)	0.063	0.625	19 (67.9)	14 (50.0)	0.063	0.563	23 (76.7)	17 (56.7)	0.031	0.344
Total cholesterol	21 (40.4)	4 (7.7)	<0.001	**<0.001**	8 (21.1)	3 (7.9)	0.227	1.000	7 (25.0)	1 (3.6)	0.031	0.313	7 (23.3)	1 (3.3)	0.070	0.633
LDL-C	21 (40.4)	11 (21.2)	0.013	0.089	13 (34.2)	2 (5.3)	0.003	**0.041**	15 (53.6)	9 (32.1)	0.070	0.563	13 (43.3)	3 (10.0)	0.002	**0.023**
HDL-C	27 (51.9)	16 (30.8)	0.013	0.089	15 (39.5)	10 (26.3)	0.302	1.000	11 (39.3)	4 (14.3)	0.016	0.172	15 (50.0)	8 (26.7)	0.143	1.000
Triglycerides	14/51 (27.5)	1/51 (2.0)	<0.001	**0.002**	7 (18.4)	2 (5.3)	0.063	0.625	11 (39.3)	0 (0.0)	<0.001	**0.012**	5 (16.7)	1 (3.3)	0.125	1.000
Testosterone	3 (5.8)	0 (0.0)	0.250	0.500	5 (13.2)	0 (0.0)	0.063	0.625	3 (10.7)	0 (0.0)	0.250	1.000	2 (6.7)	0 (0.0)	0.500	1.000
Androstenedione †	3 (5.8)	0 (0.0)	0.250	0.500	0 (0.0)	0 (0.0)	1.000	1.000	0 (0.0)	0 (0.0)	1.000	1.000	1 (3.3)	0 (0.0)	1.000	1.000
DHEAS	6 (11.5)	2 (3.8)	0.125	0.500	7 (18.4)	1 (2.6)	0.031	0.344	5 (17.9)	4 (14.3)	1.000	1.000	4 (13.3)	3 (10.0)	1.000	1.000
17-OH-progesterone	20 (38.5)	13 (25.0)	0.092	0.461	20 (52.6)	17 (44.7)	0.375	1.000	12 (42.9)	13 (46.4)	1.000	1.000	14 (46.7)	10 (33.3)	0.219	1.000

Abbreviations: BMI, body mass index; FPG, fasting plasma glucose; IRI, immunoreactive insulin; HOMA-IR, homeostasis model assessment of insulin resistance; TC, total cholesterol; LDL-C, low-density lipoprotein cholesterol; HDL-C, high-density lipoprotein cholesterol; TG, triglycerides; DHEAS, dehydroepiandrosterone sulfate; 17-OH-progesterone, 17-hydroxyprogesterone. Note: Values are presented as n (%) of participants with abnormal values. Within-group changes from baseline to 6 months were assessed using two-sided exact McNemar tests. To account for multiple testing across the examined outcomes, *p*-values were adjusted separately within each treatment group using the Holm method. Both unadjusted and Holm-adjusted *p*-values are presented, with statistical inference based on the Holm-adjusted *p*-values. For androstenedione, only values above the upper reference limit (>13.68 nmol/L) were classified as abnormal for the assessment of biochemical hyperandrogenism. Bold values indicate Holm-adjusted *p* < 0.05. For triglycerides in the metformin group, percentages were calculated from 51 participants with paired available measurements. † For androstenedione, only values above the upper reference limit were classified as abnormal, as this analysis was intended to reflect biochemical hyperandrogenism.

**Table 4:** Six-month changes in anthropometric, metabolic, lipid, and androgen parameters according to treatment group.

Parameter	Metformin Mdn [Q1, Q3]	Myo-Inositol Mdn [Q1, Q3]	Berberine Mdn [Q1, Q3]	Met + Ber Mdn [Q1, Q3]	H (3)	*p*	Post Hoc Comparisons (Dunn-Bonferroni-Adjusted *p* Values)
Δ BMI (kg/m^2^)	−4.0 [−5.1, −3.0]	0.0 [−1.0, 0.0]	−1.8 [−3.0, −1.0]	−2.0 [−2.0, −1.0]	58.19	<0.001	Metformin vs. Myo-inositol (*p* < 0.001); Metformin vs. Berberine (*p* < 0.001); Metformin vs. Metformin + Berberine (*p* = 0.001); Metformin + Berberine vs. Myo-inositol (*p* = 0.010); Berberine vs. Myo-inositol (*p* = 0.039)
Δ FPG (mmol/L)	−0.48 [−0.76, −0.12]	−0.17 [−0.39, 0.22]	0.03 [−0.34, 0.24]	−0.25 [−0.47, 0.19]	14.91	0.002	Metformin vs. Myo-inositol (*p* = 0.008); Metformin vs. Berberine (*p* = 0.010)
Δ IRI (mIU/L)	−5.64 [−13.35, −3.18]	−1.13 [−2.37, −0.21]	−1.58 [−1.96, −0.90]	−3.73 [−5.30, −2.34]	48.73	<0.001	Metformin vs. Myo-inositol (*p* < 0.001); Metformin vs. Berberine (*p* < 0.001); Metformin + Berberine vs. Myo-inositol (*p* = 0.002); Metformin + Berberine vs. Berberine (*p* = 0.017)
Δ HOMA-IR	−1.7 [−3.8, −0.9]	−0.2 [−0.6, 0.0]	−0.4 [−0.6, −0.2]	−1.1 [−1.5, −0.5]	49.24	<0.001	Metformin vs. Myo-inositol (*p* < 0.001); Metformin vs. Berberine (*p* < 0.001); Metformin + Berberine vs. Myo-inositol (*p* = 0.006); Metformin + Berberine vs. Berberine (*p* = 0.023)
Δ Total cholesterol (mmol/L)	−0.50 [−0.78, −0.18]	−0.24 [−0.56, 0.16]	−0.31 [−0.52, 0.02]	−0.42 [−0.69, 0.01]	7.38	0.061	—
Δ LDL-C (mmol/L)	−0.56 [−0.80, −0.25]	−0.30 [−0.61, 0.08]	−0.21 [−0.61, 0.13]	−0.60 [−0.94, −0.28]	7.96	0.047	No significant pairwise comparisons
Δ HDL-C (mmol/L)	0.10 [−0.02, 0.24]	0.04 [−0.06, 0.15]	0.07 [−0.06, 0.17]	0.17 [0.01, 0.24]	4.16	0.244	—
Δ Triglycerides (mmol/L)	−0.33 [−0.43, −0.14]	−0.14 [−0.27, 0.01]	−0.22 [−0.32, −0.13]	−0.15 [−0.28, 0.01]	17.59	<0.001	Metformin vs. Myo-inositol (*p* = 0.003); Metformin vs. Metformin + Berberine (*p* = 0.004)
Δ Testosterone (nmol/L)	−0.19 [−0.56, −0.05]	−0.17 [−0.43, 0.09]	−0.15 [−0.34, 0.07]	−0.21 [−0.32, 0.03]	1.83	0.609	—
Δ Androstenedione (nmol/L)	−1.33 [−2.12, −0.58]	−1.04 [−1.39, −0.38]	−0.58 [−1.23, −0.06]	−1.13 [−1.84, −0.40]	7.58	0.056	—
Δ DHEAS (µmol/L)	−1.61 [−2.38, −1.06]	−0.96 [−1.87, −0.31]	−0.49 [−1.35, 0.49]	−0.79 [−1.21, −0.24]	29.96	<0.001	Metformin vs. Myo-inositol (*p* = 0.007); Metformin vs. Berberine (*p* < 0.001); Metformin vs. Metformin + Berberine (*p* < 0.001)
Δ 17-OH-progesterone (nmol/L)	−0.27 [−0.67, 0.00]	−0.26 [−0.68, 0.12]	−0.04 [−0.29, 0.24]	−0.16 [−0.72, 0.14]	4.75	0.191	—

Abbreviations: BMI, body mass index; FPG, fasting plasma glucose; IRI, immunoreactive insulin; HOMA-IR, homeostasis model assessment of insulin resistance; TC, total cholesterol; LDL-C, low-density lipoprotein cholesterol; HDL-C, high-density lipoprotein cholesterol; TG, triglycerides; DHEAS, dehydroepiandrosterone sulfate; 17-OH-progesterone, 17-hydroxyprogesterone. Note. Values are the median [Q1, Q3] of the within-participant change (Δ = 6-month value—baseline value); negative values denote a reduction. Between-group differences were tested with the Kruskal–Wallis test followed by Dunn-Bonferroni-adjusted pairwise comparisons.

**Table 5:** Baseline-adjusted analysis of metabolic outcomes at six months.

Outcome	Myo-Inositol Adjusted Mean (95% CI)	Metformin Adjusted Mean (95% CI)	Berberine Adjusted Mean (95% CI)	Metformin + Berberine Adjusted Mean (95% CI)	Overall *p*-Value	Holm-Adjusted *p*-Value
BMI (kg/m^2^)	25.19 (24.78–25.59)	24.27 (23.84–24.69)	24.83 (24.50–25.16)	25.02 (24.77–25.28)	0.018	0.143
FPG (mmol/L)	5.14 (5.05–5.24)	5.14 (5.05–5.24)	5.16 (5.04–5.28)	5.17 (5.09–5.26)	0.957	1.000
IRI (mIU/L)	12.02 (11.20–12.85)	13.13 (12.39–13.87)	12.73 (12.08–13.39)	12.78 (11.99–13.57)	0.276	1.000
HOMA-IR	2.74 (2.52–2.95)	3.05 (2.85–3.24)	2.90 (2.69–3.11)	2.94 (2.74–3.14)	0.280	1.000
Total cholesterol (mmol/L)	4.60 (4.49–4.71)	4.52 (4.42–4.62)	4.50 (4.40–4.60)	4.50 (4.40–4.60)	0.504	1.000
LDL-C (mmol/L)	2.46 (2.33–2.60)	2.40 (2.27–2.53)	2.60 (2.43–2.78)	2.31 (2.17–2.45)	0.069	0.413
HDL-C (mmol/L)	1.42 (1.37–1.48)	1.41 (1.37–1.45)	1.41 (1.38–1.45)	1.44 (1.39–1.50)	0.808	1.000
Triglycerides (mmol/L)	1.36 (1.29–1.44)	1.25 (1.18–1.31)	1.35 (1.29–1.40)	1.37 (1.30–1.44)	0.040	0.279
Testosterone (nmol/L)	1.53 (1.40–1.67)	1.32 (1.20–1.44)	1.57 (1.47–1.67)	1.44 (1.31–1.57)	0.008	0.082
Androstenedione (nmol/L)	5.74 (5.37–6.12)	4.89 (4.48–5.30)	5.75 (5.34–6.16)	5.50 (5.12–5.88)	0.014	0.124
DHEAS (µmol/L)	8.50 (8.02–8.97)	7.46 (7.03–7.90)	9.36 (8.95–9.77)	8.73 (8.31–9.14)	<0.001	<0.001
17-OH-progesterone (nmol/L)	2.32 (2.15–2.48)	1.96 (1.80–2.11)	2.47 (2.30–2.63)	2.16 (2.02–2.31)	<0.001	0.010

Abbreviations: BMI, body mass index; FPG, fasting plasma glucose; IRI, immunoreactive insulin; HOMA-IR, homeostasis model assessment of insulin resistance; TC, total cholesterol; LDL-C, low-density lipoprotein cholesterol; HDL-C, high-density lipoprotein cholesterol; TG, triglycerides; DHEAS, dehydroepiandrosterone sulfate; 17-OH-progesterone, 17-hydroxyprogesterone; CI, confidence interval. Note: Adjusted means and 95% confidence intervals were estimated using baseline-adjusted linear regression models with robust standard errors. For each outcome, the model included the treatment group, the corresponding baseline value, age, and baseline BMI. For BMI, the model included treatment group, baseline BMI, and age. Overall *p*-values refer to the treatment-group effect. Holm-adjusted *p*-values account for multiple testing across the 12 outcome models. Bold values indicate Holm-adjusted *p* < 0.05. The triglyceride model included 147 participants because one participant had incomplete paired triglyceride measurements. Analyses were exploratory given the observational, non-randomized study design.

**Table 6:** Spearman correlations of 6-month changes—Metformin group (n = 52).

Parameter	1	2	3	4	5	6	7	8	9	10	11	12
1. Δ BMI	—											
2. Δ FPG	0.04	—										
3. Δ IRI	0.38 **	0.12	—									
4. Δ HOMA-IR	0.31 *	0.42 **	0.88 ***	—								
5. Δ Total cholesterol	−0.05	0.12	0.04	0.03	—							
6. Δ Triglycerides	0.06	0.20	0.31 *	0.25	0.24	—						
7. Δ HDL-C	−0.11	−0.27	0.05	−0.06	−0.35 *	0.00	—					
8. Δ LDL-C	0.18	0.02	0.05	0.07	−0.12	0.02	0.00	—				
9. Δ Testosterone	−0.12	−0.11	−0.01	−0.07	0.13	0.33 *	−0.03	−0.04	—			
10. Δ Androstenedione	−0.24	−0.09	0.03	0.06	0.09	0.09	−0.06	−0.16	0.01	—		
11. Δ DHEAS	−0.14	−0.19	−0.03	−0.01	0.21	0.08	−0.17	−0.01	0.28 *	0.15	—	
12. Δ 17-OHP	0.05	−0.11	−0.04	−0.02	0.12	0.00	−0.11	0.26	0.13	0.09	0.03	—

Abbreviations: BMI, body mass index; FPG, fasting plasma glucose; IRI, immunoreactive insulin; HOMA-IR, homeostasis model assessment of insulin resistance; HDL-C, high-density lipoprotein cholesterol; LDL-C, low-density lipoprotein cholesterol; DHEAS, dehydroepiandrosterone sulfate; 17-OH-progesterone, 17-hydroxyprogesterone. Note. Variables are numbered in the first column; column headers use the same numbers. Entries are Spearman ρ; * *p* < 0.05, ** *p* < 0.01, *** *p* < 0.001.

**Table 7:** Spearman correlations of 6-month changes—Myo-inositol group (n = 38).

Parameter	1	2	3	4	5	6	7	8	9	10	11	12
1. Δ BMI	—											
2. Δ FPG	0.06	—										
3. Δ IRI	0.56 ***	0.24	—									
4. Δ HOMA-IR	0.44 **	0.55 ***	0.89 ***	—								
5. Δ Total cholesterol	0.21	−0.21	0.20	0.14	—							
6. Δ Triglycerides	0.08	0.15	0.19	0.17	0.07	—						
7. Δ HDL-C	−0.34 *	−0.01	−0.23	−0.19	0.08	0.08	—					
8. Δ LDL-C	0.36 *	−0.07	0.30	0.21	0.56 ***	0.01	−0.33 *	—				
9. Δ Testosterone	0.25	0.07	0.06	0.02	0.11	−0.24	−0.36 *	0.22	—			
10. Δ Androstenedione	0.00	0.01	−0.18	−0.24	−0.13	−0.01	−0.01	−0.04	0.10	—		
11. Δ DHEAS	0.14	0.09	0.00	0.04	−0.15	−0.06	−0.20	0.14	0.29	0.22	—	
12. Δ 17-OHP	0.25	0.09	−0.03	−0.11	0.01	0.08	−0.14	0.32	0.32 *	0.41 *	0.27	—

Abbreviations: BMI, body mass index; FPG, fasting plasma glucose; IRI, immunoreactive insulin; HOMA-IR, homeostasis model assessment of insulin resistance; HDL-C, high-density lipoprotein cholesterol; LDL-C, low-density lipoprotein cholesterol; DHEAS, dehydroepiandrosterone sulfate; 17-OH-progesterone, 17-hydroxyprogesterone. Note. Variables are numbered in the first column; column headers use the same numbers. Entries are Spearman ρ; * *p* < 0.05, ** *p* < 0.01, *** *p* < 0.001.

**Table 8:** Spearman correlations of 6-month changes—Berberine group (n = 28).

Parameter	1	2	3	4	5	6	7	8	9	10	11	12
1. Δ BMI	—											
2. Δ FPG	0.16	—										
3. Δ IRI	0.22	0.09	—									
4. Δ HOMA-IR	0.20	0.69 ***	0.68 ***	—								
5. Δ Total cholesterol	0.03	0.09	0.36	0.38 *	—							
6. Δ Triglycerides	−0.12	−0.18	0.20	0.10	−0.06	—						
7. Δ HDL-C	−0.20	−0.14	−0.07	−0.17	−0.21	−0.20	—					
8. Δ LDL-C	0.01	0.11	0.31	0.20	0.26	−0.02	0.07	—				
9. Δ Testosterone	−0.03	−0.23	−0.20	−0.41 *	−0.41 *	0.10	0.11	0.01	—			
10. Δ Androstenedione	0.07	0.12	−0.15	−0.01	−0.06	0.00	−0.50 **	−0.32	−0.07	—		
11. Δ DHEAS	0.01	0.19	0.24	0.25	−0.01	−0.02	0.02	0.12	0.22	0.30	—	
12. Δ 17-OHP	−0.08	0.48 **	0.01	0.31	0.00	−0.03	−0.24	0.07	0.10	0.01	0.31	—

Abbreviations: BMI, body mass index; FPG, fasting plasma glucose; IRI, immunoreactive insulin; HOMA-IR, homeostasis model assessment of insulin resistance; HDL-C, high-density lipoprotein cholesterol; LDL-C, low-density lipoprotein cholesterol; DHEAS, dehydroepiandrosterone sulfate; 17-OH-progesterone, 17-hydroxyprogesterone. Note. Variables are numbered in the first column; column headers use the same numbers. * *p* < 0.05, ** *p* < 0.01, *** *p* < 0.001.

**Table 9:** Spearman correlations of 6-month changes—Metformin + berberine group (n = 30).

Parameter	1	2	3	4	5	6	7	8	9	10	11	12
1. Δ BMI	—											
2. Δ FPG	0.18	—										
3. Δ IRI	0.03	0.18	—									
4. Δ HOMA-IR	0.16	0.54 **	0.88 ***	—								
5. Δ Total cholesterol	0.36	0.01	0.46 *	0.35	—							
6. Δ Triglycerides	0.39 *	−0.23	0.08	0.00	0.30	—						
7. Δ HDL-C	0.12	0.01	−0.01	0.04	0.09	0.03	—					
8. Δ LDL-C	−0.10	−0.07	0.12	0.06	0.17	−0.12	0.13	—				
9. Δ Testosterone	0.19	0.26	0.00	0.11	0.10	0.18	0.32	−0.18	—			
10. Δ Androstenedione	0.29	0.30	0.08	0.19	−0.04	0.17	−0.29	−0.51 **	0.17	—		
11. Δ DHEAS	−0.04	−0.11	0.48 **	0.39 *	−0.04	0.03	−0.03	−0.02	0.26	0.21	—	
12. Δ 17-OHP	0.12	0.22	0.03	0.08	0.17	−0.03	0.03	−0.02	0.08	0.24	−0.09	—

Abbreviations: BMI, body mass index; FPG, fasting plasma glucose; IRI, immunoreactive insulin; HOMA-IR, homeostasis model assessment of insulin resistance; HDL-C, high-density lipoprotein cholesterol; LDL-C, low-density lipoprotein cholesterol; DHEAS, dehydroepiandrosterone sulfate; 17-OH-progesterone, 17-hydroxyprogesterone. Note. Variables are numbered in the first column; column headers use the same numbers. n = 30. * *p* < 0.05, ** *p* < 0.01, *** *p* < 0.001.

## Data Availability

The data supporting the findings of this study are available from the corresponding author upon reasonable request. The data are not publicly available due to privacy and ethical restrictions related to patient data.
